# Retracted: N-(1-Pyrenyl) Maleimide Induces Bak Oligomerization and Mitochondrial Dysfunction in Jurkat Cells

**DOI:** 10.1155/2021/9835474

**Published:** 2021-10-28

**Authors:** BioMed Research International

*BioMed Research International* has retracted the article titled “N-(1-Pyrenyl) Maleimide Induces Bak Oligomerization and Mitochondrial Dysfunction in Jurkat Cells” [1] due to concerns regarding the reliability of the results. The journal has been made aware [2] that the article includes nucleotide sequences which appear to be incorrectly targeted, as follows:

- CTTCGTACCATTTTTG targets ZHX3 mRNA, not Bak as described in the article

- TGTGTGAAAGTTTTTG targets YWHAEP7 non-coding RNA, not Bid as described in the article

- TTGGCAACCGCTTTTTG targets Luciferase 1 in P. pyralis, but only with 93% identity

The authors did not respond to our requests for clarification, and due to the above concerns, it is being retracted with the agreement of the journal and the editorial board.
